# Different Representation Procedures Originated from Multivariate Temporal Pattern Analysis of the Behavioral Response to Pain in Wistar Rats Tested in a Hot-Plate under Morphine

**DOI:** 10.3390/brainsci9090233

**Published:** 2019-09-12

**Authors:** Maurizio Casarrubea, Stefania Aiello, Andrea Santangelo, Giuseppe Di Giovanni, Giuseppe Crescimanno

**Affiliations:** 1 Laboratory of Behavioral Physiology, Department of Biomedicine Neuroscience and Advanced Diagnostics, Human Physiology Section “Giuseppe Pagano”, University of Palermo, Palermo 90133, Italy; stefania.aiello@unipa.it (S.A.); a.santangelo@ymail.com (A.S.); giuseppe.crescimanno@unipa.it (G.C.); 2 Laboratory of Neurophysiology, Department of Physiology and Biochemistry Faculty of Medicine and Surgery, University of Malta, Msida 2080, Malta; giuseppe.digiovanni@um.edu.mt

**Keywords:** pain, T-pattern analysis, T-pattern, multivariate analyses, hot-plate, morphine

## Abstract

Temporal pattern analysis is an advanced multivariate technique able to investigate the structure of behavior by unveiling the existence of statistically significant constraints among the interval length separating events in sequence. If on the one hand, such an approach allows investigating the behavioral response to pain in its most intimate and inner features, on the other hand, due to the meaning of the studies on pain, it is of relevant importance that the results utilize intuitive and easily comprehensible ways of representation. The aim of this paper is to show various procedures useful to represent the results originating from the multivariate T-pattern analysis of the behavioral response to pain in Wistar rats tested in a hot-plate and IP injected morphine or saline as a control.

## 1. Introduction

Behavior consists of patterns in time thus, as suggested by Eibl-Eibesfeldt, investigations of behavior must necessarily deal with sequences that are not easily perceivable [1]. Such an aspect calls for improved means of detection and data analysis. Using a multivariate technique known as T-pattern analysis (TPA) [2,3,4], it is possible to detect recurring sequences of events characterized by statistically significant constraints among the interval length separating them. Such a multivariate approach has been fruitfully applied during the last decades to study several aspects of animal and human behavior. For instance, concerning animal subjects, TPA has been applied to study cognitive performances in starlings [5], courtship behavior in insects [6], compulsive behaviors in mice [7,8], patterns of swimming in cods [9], social behavior in wolves [10] and rats [11], or anxiety-related behavior in rats [12,13,14]. As to human subjects, T-patterns have been utilized to study patterns of interaction in autism disorders [15], self-injurious behavior in psychiatric patients [16,17], social interactions in children [2], patterns in communication and language [18], sport, and/or physical activity [19,20,21]. 

The hot-plate test is a well-known model of supraspinal pain [22]. By utilizing such an approach it becomes possible to assess, in rodents, not only simple responses to pain but also complex behaviors supraspinally organized [22]. Probably one of the most significant drawbacks of hot-plates in the study of pain-related behaviors is the necessarily short observation window. During the hot-plate test, indeed, the animal is allowed to freely explore the heated perimeter for a minimal amount of time, e.g., 10 s [23,24], to avoid serious tissue injuries. Such a short observation period is, probably, the main reason that dampened the complex analyses of rodent’s behavior in this experimental apparatus. As a matter of fact, during the last three decades most studies with a hot-plate have used the evaluation of few parameters of isolated behavioral elements such as dose-response curves, latencies, or durations of individual responses to pain (e.g., paw-licking, stamping, or jumping), detached from the comprehensive behavioral structure. On the other hand, only a few works have used a different approach, i.e., transition matrices and related elaborations, e.g., see [25,26,27,28] and TPA [24]. Particularly, through TPA it is possible to obtain detailed information on the behavioral structure. In addition, TPA presents significant advantage if compared with more conventional multivariate approaches based on transition matrices e.g., see [25,26,27,28]. Namely, the possibility to assess the animal’s real-time behavior with an unrivaled level of detail. The knowledge of the real-time structure of behavior following nociceptive stimulation in the hot-plate test is able to offer new and interesting topics of discussion concerning pain-related behavioral characteristics and the approaches that must be used for their study.

The aim of this paper is, therefore, to present an overview of the graphical representations useful to illustrate real-time occurrences of T-patterns in rats observed in the hot-plate test. In the following two sections a brief overview concerning T-pattern analysis will be presented. 

## 2. Materials and Methods

### 2.1. T-pattern Analysis

A more detailed description of theories and concepts behind TPA and related applications both in human and animal subjects can be found in our reviews [4,29] and in our recent book [30]. TPA is a multivariate technique carried out using a specific software tool known as THEME^TM^ (PatternVision Ltd, Reykjavik, Iceland). Simply stated, TPA allows the detection of repetitive aspects of a given behavior [2,3,4].

A T-pattern can be expressed using this text expression:

X_1_ ≈ dt_1_ X_2_ ≈ dt_2_ X_3_… X_i_ ≈ dt_i_ X_i+1_… X_m−1_ ≈ dt_m−1_X_m_,

where X_1_ … X_2_ … Xm terms designate the events in sequence and ≈dt indicates the temporal distances between these events. Thus, the term X_i_ ≈ dt_i_ X_i+1_ indicates that the event X_i_ is followed dt_i_ (≥0) time units later by the following event X_i+1_ and, importantly, that these events are separated by a statistically invariant time distance ≈dt_i_. These temporal distances among the events represent the targets of the software’s detection system. For example, given an observation period t0-tX encompassing numerous repeated instances of a number of events, such as “A”, “B”, “C”, …“X”,“Y”, “Z”, etc. In a first step, the software compares the distributions of each pair of the events, e.g., “A” and “B”, searching for a time interval so that, more often than chance expectation, A is followed by B within that interval. If such a circumstance is verified, then A and B do represent a T-pattern encompassing two events and indicated as (A B). In a second phase, such a first level pattern is considered as A or B terms to detect higher patterns, e.g., ((A B) C). Thus, more complex patterns may be formed on the basis of such a hierarchical bottom-up detection process. When no more patterns are found, the search stops.

### 2.2. Representations and Illustrations of T-Patterns

As summarized in the previous section, TPA is a technique able to detect sequences of events characterized by significant constraints on the interval length separating them. This analysis can be considered in terms of a special multivariate hierarchical clustering technique with critical interval detection providing a kind of similarity [3]. Albeit TPA is, taking into consideration its underlying concepts and theory, very different from the largest amount of existing multivariate approaches, it has probably an important common aspect with all the existing multivariate techniques: The need to represent results in a straightforward fashion. This is an issue often underestimated in behavioral studies employing multivariate approaches. An example, in this sense, concerns a well-known multivariate approach based on transition matrices elaboration, i.e., the adjusted residuals analysis. Adjusted residuals are transitions occurring significantly less or significantly more often than expected. Though adjusted residuals can be expressed employing a transition matrix, this representation is difficult to interpret even for experienced readers. A transition matrix is, indeed, a table filled with hundreds or even thousands of numbers and, as such, is very difficult to understand even for very skilled eyes. On the contrary, adjusted residuals can be expressed, more intuitively, using path diagrams where the thicknesses of the arrows refer to the value of the adjusted residuals in the transition matrices [31,32]. Accordingly, the adjusted residual matrix becomes immediately appreciable even at a very first glance. The TPA is not based, as mentioned above, on the analysis of matrices, but just like the adjusted residuals, its results might be expressed through dozens of tables containing a lot of quantitative information. This kind of approach should be avoided especially when results originating from experiments on pain modulation need to be clearly and visually illustrated.

### 2.3. Subjects and Apparatus

Subjects and housing: 20 adults, male, specific pathogen-free, Wistar rats (Harlan, Italy), 60 ± 5 days old, weighing 220–250 g, were employed. Animals were housed in a room maintained at a constant temperature of 23 ± 1 °C, under a 12-h light/dark cycle (lights on 07:00 a.m., lights off at 07:00 p.m.). Standard laboratory pellets and water were freely available.

Apparatus: The hot-plate apparatus consisted of a solid aluminum plate heated and maintained at the constant temperature of 54 ± 0.5 °C. Such a temperature was constantly monitored by an on-board module with a digital display. The heated surface was confined by a transparent removable Plexiglas cylinder (20 cm diameter, 40 cm height).

### 2.4. Procedure

All the experimental observations were carried out from 09:00 a.m. to 12:00 a.m. To minimize possible transfer effects and to avoid potential olfactory and/or visual influences, animals were moved from the housing room to the testing room inside their own home cages and allowed to acclimatize for 30 min far from the hot-plate apparatus. In 10 subjects, randomly selected, morphine (Monico SPA, Italy) was administered IP at a dose of 12 mg/kg in a volume of 1 mL saline. This dose was selected, on the basis of our previous studies [23,24], because it has been demonstrated to be able to induce excellent analgesic effects without impairing locomotor activities [23,24]. A volume of 1 mL of saline was injected in the 10 remaining subjects, used as a control group. After the acclimation period, each animal was placed on the heated surface. All subjects were tested 30 min after IP injections. To avoid tissue injury, each animal was removed from the apparatus after 10 s. Animals were experimentally naive and tested only once. After each observation, the cylinder and the plate surface were carefully cleaned with ethylic alcohol (70%) to remove possible scent cues left by the preceding animal. Rodent behavior was recorded by means of a digital camera (Toshiba HD-DV camcorder model P10) placed in front of the hot-plate apparatus and digital video files were stored in a personal computer for the following analyses.

### 2.5. Data Analysis

**Figure 1:**
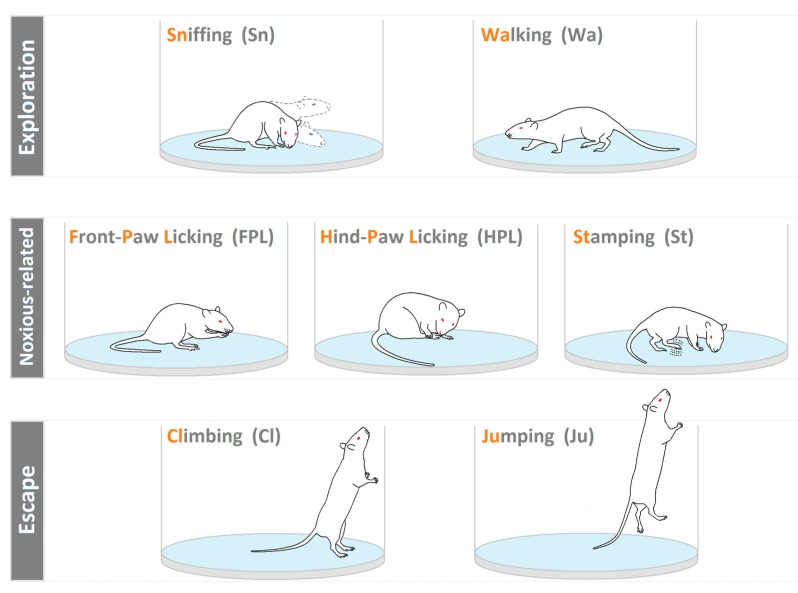
Ethogram of rat behavior. Exploration components = sniffing (Sn): The rat smells the environment, moving the vibrissae and/or the head; and walking (Wa): The rat walks around sniffing the ground and/or the Plexiglas cylinder. Noxious-related components = front paw licking (FPL): The rat licks its forepaws; hind paw licking (HPL): The rat turns its head toward its back paws and licks one of them; and shaking/stamping (St): A paw is rapidly shaken and/or stamped on the ground. Escape components = climbing (Cl): The rat leans against the Plexiglas cylinder; and jumping (Ju): The rat leaps off the surface, trying to pass over the Plexiglas cylinder.

Generally, one of the first steps in a behavioral study is the description of the behavioral categories utilized, that is, the so called ethogram. This aspect, often underestimated, is of the highest importance because, simply stated, it represents the link between the behaviors, object of the research, and what the reader will be able to understand, on the basis of the descriptions provided. For such a reason, a preliminary step should be the illustration of all the components of the behavioral repertoire. In the present study, video files, recorded during experimental sessions, were analyzed using a personal computer and a professional software coder (The Observer, Noldus IT, The Netherlands). On the basis of our previous studies [23,24,33], a rat’s behavior in a hot-plate was classified in three main categories of behavioral components (ethogram in Figure 1): Exploratory components: Sniffing (Sn), walking (Wa); primary noxious-related components: Front paw licking (FPL), hind paw licking (HPL), and shaking/stamping (St); escape components: Climbing (Cl), and jumping (Ju). 

On the basis of these categories, the behavior of each subject was manually annotated by a highly trained observer, blind to treatment. After such an annotation process was completed, event log files for each subject were stored in a personal computer. Event log-files were then imported in Theme software for T-patterns detection and analysis. The results are presented in terms of terminal strings (that is, a table with textual representation of events in sequence within parenthesis indicating the bottom-up detection level), length distribution histogram (that is, a histogram containing number of different T-patterns detected for each length), terminal strings with associated tree structure, and, finally, terminal strings with associated raster-plot indicating the onset of each detected pattern during the observation time window. The output of the Theme software for one T-pattern will be also presented.

### 2.6. Statistics

Event log files containing hundreds or even thousands of events, depending on the category table utilized, are potentially able to generate an enormous amount of possible relationships among the events. Such a simple consideration raises an important issue, which is whether T-patterns have been detected only by mere chance. To exclude this possibility, Theme software performed 10 randomizations of the original components annotated for each animal in their respective event log files; then these data were re-analyzed; finally, the mean number of patterns from the analysis of randomized data was compared with patterns detected in the original non-randomized datasets. As to the comparison of percent distributions of T-patterns containing individual components of the behavioral repertoire, Fisher’s exact probability test was utilized.

### 2.7. Ethical Statement 

All procedures involving the use of animals were carried out in accordance with the European Communities Council Directive (2010/63/EU) and approved by the official Veterinary Committee appointed by the University of Palermo, Italy.

## 3. Results

Table 1 presents all the T-patterns detected in both groups in terms of terminal strings and related occurrences, that is, the overall number of occurrences of each T-pattern. Overall, 18 different T-patterns, occurring 147 times were detected in the saline group; only one T-pattern occurring nine times was detected in the morphine group.

**Figure 2:**
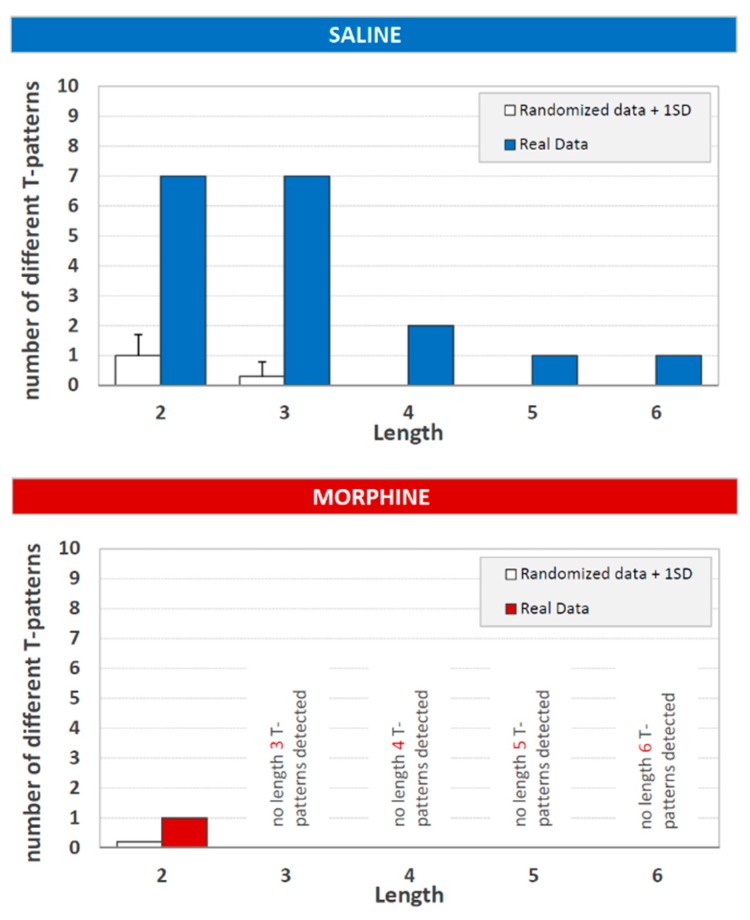
T-pattern length distribution in real data (filled bars) and randomized data + 1 SD (white bars), in control (saline) and morphine (12 mg/kg in a volume of 1 mL of saline) injected groups. X-axis = T-pattern length, i.e., number of events in the T-pattern’s structure; and Y-axis = number of different T-patterns detected. Data obtained from the analysis of two groups each encompassing 10 subjects.

The length distribution of detected T-patterns in both groups is presented in Figure 2. As to the saline group, seven different T-patterns contained two events in sequence, seven three events, two four events, one five events, and, finally, one pattern did contain a sequence of six events. As to the morphine group, only one T-pattern containing two events in the sequence was detected.

**Figure 3:**
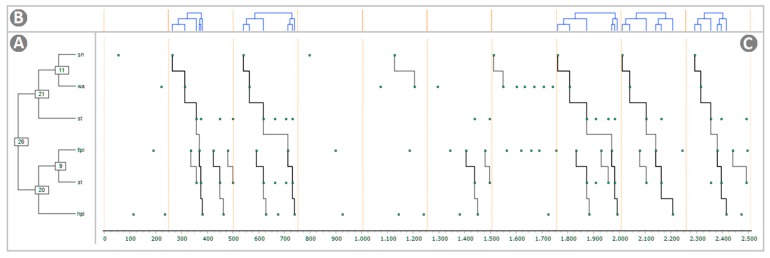
Visualization of the longest T-pattern detected in both groups, as provided by Theme software. (**A**) Static detection tree, that is, the level-by-level detection of the currently selected pattern; each terminal event is aligned with its occurrences, indicated by dots in (**C**) - numbers in boxes are identification codes of the selected pattern and lower level sub-patterns; (**B**) Dynamic detection tree, that is, the same tree structure presented in (**A**) but rotated so that each terminal is temporally synchronized and aligned with the events (dots) reported in the connection diagram below; (**C**) Connection diagram, that is, events (dots) connected by means of lines forming the recurrent pattern; X-axis = time in milliseconds. For abbreviations, see Figure 1.

**Figure 4:**
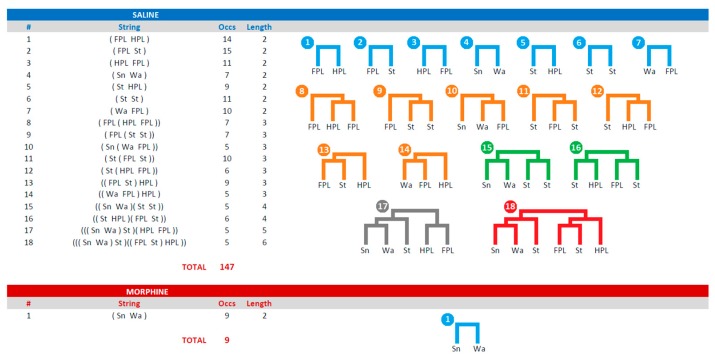
Results of T-pattern detection in terms of terminal strings (events in brackets) and corresponding static tree structures. Progressive numbers on the left of each string indicate the corresponding tree structure illustrated on the right side of the panel. Numbers on the right side of each string indicate overall occurrences (Occs) and length (i.e., number of events in T-pattern’s structure). For illustrative purposes, the length of each pattern is presented using different colors. Data obtained from the analysis of two groups each encompassing 10 subjects. For abbreviations, see Figure 1.

**Figure 5:**
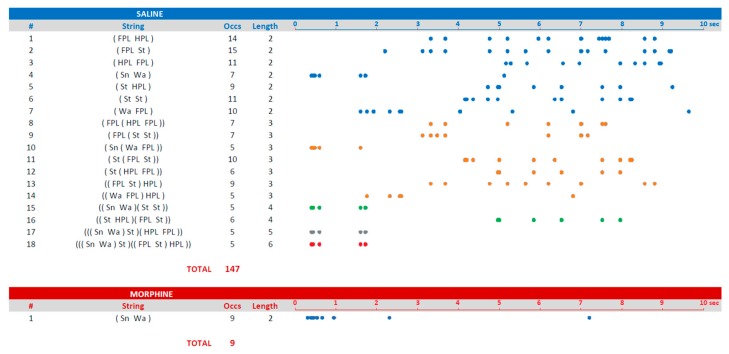
Results of T-pattern detection expressed as terminal strings (events in brackets) and the corresponding raster-plot. Dots indicate the onset of the first event of each T-pattern (see Figure 4). Numbers on the left of each row indicate the corresponding T-pattern presented in Figure 4. For illustrative purposes, pattern length is presented using the same colors as in Figure 4. X-axis = time in seconds. Data obtained from the analysis of two groups each encompassing 10 subjects. For abbreviations, see Figure 1.

The graphical output of Theme software for the selected T-pattern #18 from the saline group (see Table 1) is illustrated in Figure 3. The terminal strings, as presented in Table 1, and their static tree structures are illustrated in Figure 4. The terminal strings, as presented in Table 1, and a related raster-plot illustrating the onset of each T-pattern, are illustrated in Figure 5.

**Figure 6:**
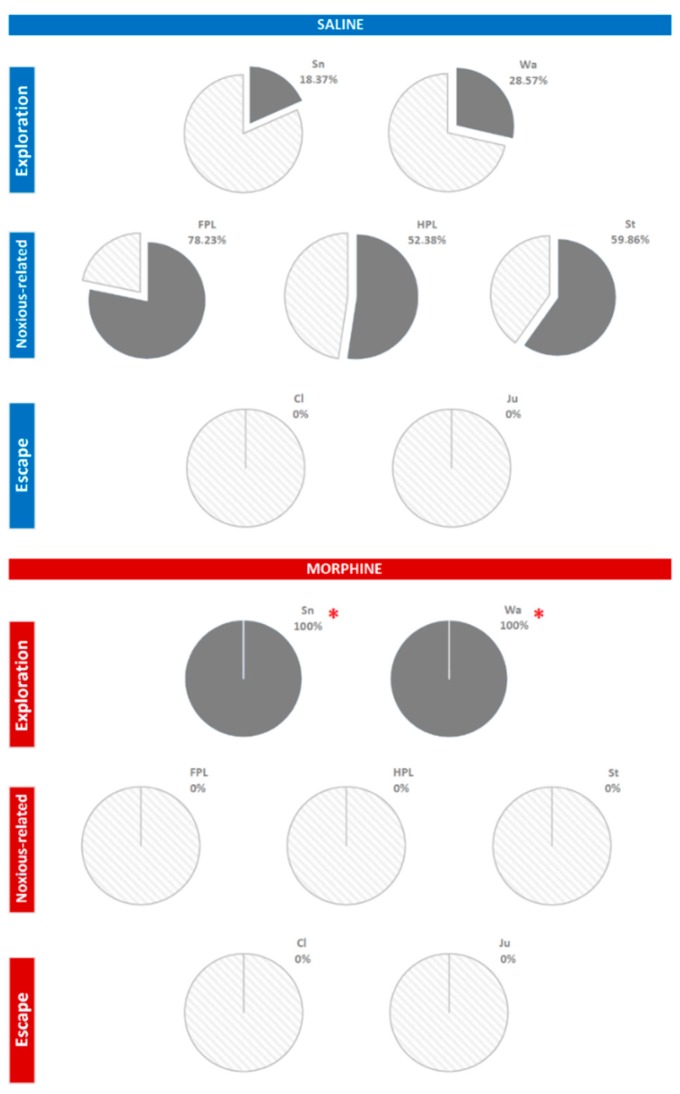
Pie charts indicating the percent of T-patterns containing each component of the behavioral repertoire. * = significant (*p* < 0.0001) difference between morphine and saline, as assessed by the Fisher’s exact probability test. Data obtained from the analysis of two groups each encompassing 10 subjects. For abbreviations, see Figure 1.

Finally, the percent distribution of T-patterns containing each component of the behavioral repertoire is shown in Figure 6. As to the saline group, sniffing was present in 18.37% of T-patterns detected, walking in 28.57%, front paw licking in 78.23%, hind paw licking in 52.38%, and stamping in 59.68%. No escape components were detected. As to the morphine group, 100% of T-patterns encompassed sniffing and walking. No T-patterns containing noxious-related and escape components were found. Fisher’s exact probability test revealed highly significant (*p* < 0.0001) differences between percent distribution of T-patterns containing walking and sniffing in the two groups.

## 4. Discussion

The present study showed the usefulness of multivariate T-pattern analysis in evaluating the behavior of rats in the hot-plate and the best graphical approaches to highlight possible changes induced by the administration of independent variables such as morphine.

### 4.1. Ethogram and Related Illustrations

The first step in the study of a given behavioral response, and in particular of the one induced by noxious stimuli, was based on the organization of a valid ethogram. By definition, an ethogram is a list containing the formal description of the various behavioral elements that the subject performs during the test. The organization of the ethogram, originating from the observation of the responses to pain, is of high relevance since the illustration of each behavior greatly improves the understanding of the results obtained from TPA. In this paper, we reported a detailed description of the rat behavioral repertoire on the hot plate. The duration of the exposure to the hot surface, not more than 10 s, was due to the need to avoid tissue damage to the animal. Once placed on the hot-plate surface, the animal will carry out behaviors that orbit around the salience of the noxious stimulus, i.e., the temperature and the area available to organize the behavioral response.

### 4.2. Terminal Strings

After a T-pattern search procedure was completed, the following step was to export all the T-patterns detected in the data set. Accordingly, it was possible to export the different T-patterns in the form of terminal strings (Table 1). These strings were representations where different events in sequence were enclosed in brackets indicating the hierarchical order in which they were identified during the detection process. For example, T-pattern # 2 (FPL St), including only the two events indicated, occurred 15 times. In turn, this sequence of two events was significantly followed up to nine times by the HPL event, as indicated by the T-pattern # 13 ((FPL St) HPL). The inner parentheses indicated the simplest 2-event T-pattern (FPL St), while the HPL coupling was indicated by two parentheses spanning the entire pattern, and so on, up to any level. This representation was certainly useful because it highlights the structure of the T-patterns found, their length and how many times they occur.

### 4.3. Length Distribution

During the T-pattern search run, the software analyzed and compared the distributions of all the events in the processed data set evaluating the existence of significant distance among all the events in sequence. For instance, given “A” and “B” two hypothetical events, the algorithm assessed the existence of a time interval so that “A” was followed by “B” within that interval; in an affirmative instance, A and B would represent a T-pattern encompassing two events and indicated as (A B), that is, a simple two-events T-pattern. In a second step, such a first level pattern was considered as potential “A” or “B” terms to detect higher-order patterns, e.g., ((A B) C), ((A B)(C D)), etc., and so forth, up to any level. Thus, after such a search process was completed, various structurally different T-patterns encompassing a variable number of events would be detected, depending on the data set complexity: Two events, three events, four events, and so on. In present results, the detection process had revealed 18 different T-patterns in the saline group: Seven different T-patterns contain two events in sequence, seven three events, two four events, one five, and, finally one six events. The representation of the length distribution of the detected T-patterns utilizing the histogram was, therefore, a further important step because it was able to provide a synopsis on two of the most important aspects of T-patterns detected in the response to pain: Their complexity (i.e., the number of events in sequence) and their variability (i.e., the number of different sequences detected). Notably, length distribution contained also the results of the detection process performed on randomized data (Figure 2, empty bars; see Section 2.4).

### 4.4. Static Trees, Dynamic Trees, and Connection Diagram

A detected T-pattern is illustrated in Figure 3. This is, by far, the most complete representation of T-pattern detection. The image consisted of three different panels strictly related. The static detection tree on the left (Figure 3A) represents the level-by-level detection of the given pattern (in this example, saline group, TP#18) so that each terminal event was aligned with its occurrences (dots in panel C). The dynamic detection tree on the top (Figure 3B) was, basically, the tree structure in panel A but rotated so that each terminal was aligned with the events (dots in panel C) reported in panel below. Finally, the connection diagram (Figure 3C), encompassed events (dots) occurring at the specific time point (X-axis) connected by means of lines forming the recurrent pattern (here occurring five times). Notably, the graphical output was highly customizable and almost every detail of the image could be modified by means of various and useful options. In detail, in Panel A it was possible to modify font size, font color, connections colors, and their width; in Panel B it was possible to modify the color of trees and to add an X-axis (time) and a Y-axis (level of detection). Finally, in Panel C, it was possible to modify point color, point size, line color, and width. Such an illustrative approach is highly useful because it helps to understand the relationships linking the different behavioral events induced by the noxious stimulus, how events are connected, and, of course, the real-rime structure of the given pattern. As mentioned in Section 2.4, our results concerned of 10 s observations that were, taking into consideration such a brief observation period, quite short event log files. Even so, 18 images similar to Figure 3 for saline and one for the morphine group would be necessary. Actually, it is not uncommon to detect higher amounts of T-patterns. For instance, we have detected in two groups of rodent strain, tested in the elevated plus-maze, 13 and 21 different t-patterns [13]; notably, the situation in terms of high number of T-patterns detected can be even worst: For instance, in rats under standard vs. hyperglycidic diet, 50 and 703 different T-patterns were respectively detected [34]. In these circumstances, because of the large amount of necessary space, we had chosen the approach that represents the results of T-pattern analysis using strings, static trees, and raster-plots, the latter indicating the onset of each pattern [13]. 

### 4.5. Strings, Trees, and Raster-Plots

Since the number of different patterns is not exceedingly high, as in present results, it is possible to consider the utilization of an illustration presenting, at the same time, the strings and related static trees (Figure 4). The advantage of such an approach lies in the intuitive representation offered by dendrogram-like trees. Each tree must be labeled with the number indicating the corresponding string in the left column. Besides, each tree, for illustrative purposes, can be presented in a different color. For example, in Figure 4, t-patterns containing two events are in blue, three events in orange, four in green, and so on. Taking into consideration the amount of space required, it is clear that about 50 or 60 different T-patterns can be represented using this method. However, the main problem of this representation is that static trees are detached from the temporal context. Figure 4 illustrates the strings, their overall occurrences, their lengths, and their trees, but not their occurrence in real-time. To address such an important issue, we have developed the representation of T-patterns by means of raster-plots that is, a diagram illustrating the onset of each T-pattern. To arrange T-patterns’ raster-plots it is essential to export the start-time of each T-pattern by using the appropriate option available in the software we used. After that, once the onset of each detected pattern is available, a common datasheet program will be able to create such a representation with dots (Figure 5) or bars [13,35] indicating the onset of each pattern, that is, the beginning of the first event in sequence. Raster-plots, properly organized, are much less demanding in terms of space required. In addition, this is a useful representation because it makes available an overview on the distribution of sequences within the comprehensive observation period. Notably, taking into consideration the extremely limited period of observation required by the hot-plate test (because of the heated surface, to avoid tissue injuries), the representation by means of raster-plots can be very useful to detail the real-time onset of each sequence of events. For instance, concerning present results, even at a very first glance, Figure 5 highlights that, in the saline group, T-patterns containing sniffing (Sn) and walking (Wa) were the first to appear (TP# 4,10,15,17,18), rapidly followed by t-patterns containing components evoked by noxious stimulation; on the other hand, after morphine administration, only an initial exploration was present, mainly within the first second, not followed by patterns containing noxious evoked components. This evidence is consistent with the well-known analgesic properties of morphine. 

### 4.6. Percent Composition of T-Patterns 

To illustrate the composition of T-patterns, including each component of the behavioral repertoire as percent distribution, pie charts may represent a useful option. On this subject, pie charts are able to provide an intuitive point of view on behavioral architecture. For example, pie charts in Figure 6 show that T-patterns detected in the saline group were composed of exploration and noxious-related components; on the other hand, morphine removed behavioral sequences related to noxious stimulation and promoted the occurrence of T-patterns 100% containing exploratory components.

### 4.7. Translational Perspectives

Due to the increasing amount of studies on the neurophysiological characteristics of pain and on the efficacy of its pharmacological treatment, it could be of current help to represent results, originating from both human and animal research, in a clear and fast to understand way. Recent work by Hoegh et al. [36], reported delayed effects of attention on pain sensitivity and conditioned pain modulation. In this study, attentive condition and conditioning pain modulation were demonstrated to modulate pain in a healthy man. A further closer examination of the interesting results may lie in T-pattern analysis and its graphical representation. Another starting point to consider the use of T-pattern analysis in humans being origins from the reviews of Gulur and Nelli [37] on persistent postoperative pain. They suggest that the understanding of the transition from acute to chronic pain is essential to both prevent and manage this widespread post-surgical condition. The identification of a temporal organization of the behavioral response to noxious stimuli both in the acute and chronic condition could help in identifying suitable pharmacological preventing treatments. 

## 5. Conclusions

In conclusion, T-pattern analysis could be considered a useful approach to illustrate the temporal structure of the behavioral response to pain and its modulation both in humans and rodents. Moreover, different graphical representation procedures, covering nearly almost every need in terms of number of T-patterns detected, it is suggested could improve clearness and usefulness of the results.

## Figures and Tables

**Table 1:** Results of T-pattern detection in both groups of rats (morphine: 12 mg/kg IP in a volume of 1 mL of saline; saline: Volume of 1 mL) expressed as terminal strings (events in brackets). Progressive numbers on the left of each string indicate the corresponding string’s identification number. Numbers on the right of each string indicate overall occurrences (Occs) and length (i.e., number of events in the T-pattern’s structure). Data obtained from the analysis of two groups each encompassing 10 subjects. For abbreviation see Figure 1.

#	String	Occs	Length
**Saline**			
1	(FPL HPL)	14	2
2	(FPL St)	15	2
3	(HPL FPL)	11	2
4	(Sn Wa)	7	2
5	(St HPL)	9	2
6	(St St)	11	2
7	(Wa FPL)	10	2
8	(FPL (HPL FPL))	7	3
9	(FPL (St St))	7	3
10	(Sn (Wa FPL))	5	3
11	(St (FPL St))	10	3
12	(St (HPL FPL))	6	3
13	((FPL St) HPL)	9	3
14	((Wa FPL) HPL)	5	3
15	((Sn Wa)(St St))	5	4
16	((St HPL)(FPL St))	6	4
17	(((Sn Wa) St)(HPL FPL))	5	5
18	(((Sn Wa) St)((FPL St) HPL))	5	6
	Total	147	
**Morphine**			
1	(Sn Wa)	9	2
	Total	9	
